# Fermentation Supernatant of Elderly Feces with Inulin and Partially Hydrolyzed Guar Gum Maintains the Barrier of Inflammation-Induced Caco-2/HT29-MTX-E12 Co-Cultured Cells

**DOI:** 10.1021/acs.jafc.2c06232

**Published:** 2023-01-09

**Authors:** Gaku Kono, Kazuma Yoshida, Eri Kokubo, Masayuki Ikeda, Takeshi Matsubara, Takahiro Koyama, Hiroshi Iwamoto, Kazuhiro Miyaji

**Affiliations:** Health Care and Nutritional Science Institute, Morinaga Milk Industry Co., Ltd., 1-83, 5-Chome, Higashihara, Zama, Kanagawa Prefecture 252-8583, Japan

**Keywords:** intestinal barrier, gut microbiota, fecal fermentation, prebiotics, inulin, PHGG

## Abstract

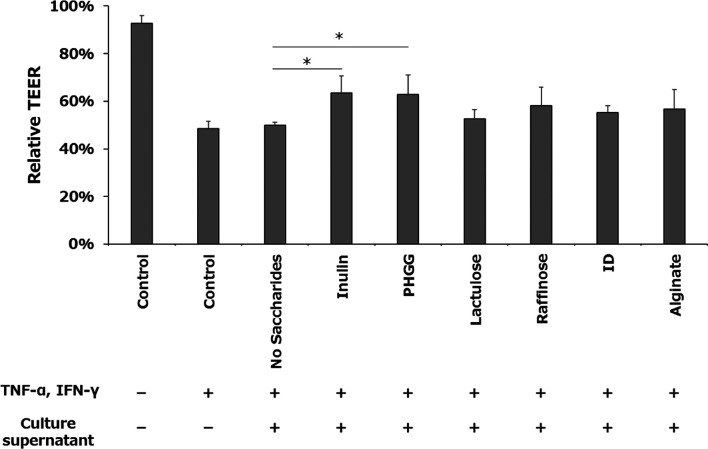

Intestinal barrier function declines with aging. We evaluated the effect of dietary fibers and indigestible oligosaccharides on intestinal barrier function by altering the microbiota of the elderly. The feces were anaerobically cultured with indigestible dextrin, inulin, partially hydrolyzed guar gum (PHGG), lactulose, raffinose, or alginate, and the fermented supernatant was added to inflammation-induced Caco-2/HT29-MTX-E12 co-cultured cells. Our data showed that inulin- and PHGG-derived supernatants exerted a protective effect on the intestinal barrier. The protective effect was significantly positively correlated with total short-chain fatty acids (SCFAs) and butyric acid production in the supernatant and negatively correlated with the claudin-2 (*CLDN2*) gene expression in the cultured cells. Furthermore, we showed that the *CLDN2* levels are regulated by butyric acid. Thus, inulin and PHGG can change the intestinal environment of the elderly and maintain the intestinal barrier by accelerating the production of SCFAs and modifying the expression levels of barrier function-related genes.

## Introduction

Aging changes the gut microbiota significantly; for instance, it reduces *Bifidobacterium* and *Faecalibacterium* and increases *Bacteroides*.^1−4^ These changes in the microbiota alter the metabolites in the intestines of the elderly,^3,5^ and this may also affect their intestinal barrier function.^6^ A study comparing the blood zonulin levels in the elderly (70 years or older) and young (18–30 years) individuals showed an increase in zonulin levels in the elderly, thereby indicating reduced intestinal barrier function in the elderly.^7^ Increased intestinal permeability causes bacterial translocation and influx of toxins such as lipopolysaccharide (LPS) into the body, which causes systemic inflammation and metabolic dysfunction, leading to the induction and worsening of various diseases, such as liver disease and diabetes mellitus.^8,9^ Therefore, elderly people need to pay attention to maintaining the intestinal environment and intestinal barrier function.

Intestinal barrier dysfunction is associated with increased levels of pro-inflammatory cytokines, such as tumor necrosis factor-alpha (TNF-α) and interferon-gamma (IFN-γ), and indeed, these pro-inflammatory cytokines are increased in the elderly and prevalent individuals with a reduced intestinal barrier.^10,11^ These cytokines affect the expression levels of several tight junction genes; for example, TNF-α increases the expression level of *claudin-2* (*CLDN2*) or decreases that of *zonula occluden-1* (*ZO-1*), which in turn increases intestinal permeability.^12,13^

Dietary fibers and some oligosaccharides are not digested or absorbed by the human gastrointestinal tract and are expected to exert beneficial effects by changing the microbiota or enhancing the intestinal barrier, which is mediated by metabolites derived from intestinal bacteria.^14,15^ These are called prebiotics.

Numerous clinical studies have evaluated dietary fiber or indigestible oligosaccharide content for intestinal barrier protection.^16−18^ However, only few studies have focused on the elderly, who need special attention in terms of the intestinal barrier, and the evaluated prebiotics are limited. The microbiota of the elderly is different from that of the young; therefore, the dynamics of the microbiota and the production of metabolites can differ between the young and elderly when prebiotics are ingested. Therefore, previous research on the intestinal barrier in the young does not necessarily apply to the elderly. In vitro studies have also evaluated the effect of prebiotics on the intestinal barrier using cultured intestinal cells.^19,20^ However, some studies have evaluated this by adding prebiotics directly to the cells, without considering the prebiotic function of changing the gut microbiota and the metabolism of intestinal bacteria. Prebiotics affect the intestinal barrier more due to the metabolites produced by intestinal bacteria than directly by the prebiotics.^6^ Therefore, in vitro studies should try to mimic biological conditions more precisely. Recently, a pH-controlled fermentation system for human gut microbiota was established, and this in vitro system enabled the evaluation of the effects of prebiotics on the microbiota and their metabolites, reflecting the communication of the entire intestinal bacteria.^21^

This study aimed to evaluate whether dietary fibers and indigestible oligosaccharides, known as prebiotics, protect the intestinal barrier from inflammation-induced damage by altering the metabolites of microbiota in the elderly. Therefore, we cultured inulin, partially hydrolyzed guar gum (PHGG), lactulose, raffinose, indigestible dextrin, and alginate using a pH-controlled fermentation system^21,22^ with feces collected from the elderly and evaluated the effects of the culture supernatants on the intestinal barrier function followed by an inflammation-induced epithelial cell assay (Caco-2 and HT29-MTX-E12 co-culture system). Additionally, we analyzed the relationship between the protective effect of prebiotics on the intestinal barrier and the metabolites in the fecal fermented supernatant and gene expression levels of cells involved in the intestinal barrier.

## Materials and Methods

### Fecal Sample Collection

Fecal samples were collected from healthy adult Japanese volunteers. Subjects were excluded if they were suspected of having infectious bowel disease. The protocol for sample collection and handling was approved by the Ethics Committee of the Japan Conference of Clinical Research (Tokyo, Japan; protocol code, NFSFA-01; date of approval, October 18, 2019). Informed consent was obtained from all the participants. Fecal samples were collected from four volunteers, two men and two women, with a mean age of 75.5 years (68–85 years). Their dietary habits were assessed using a brief-type self-administered diet history questionnaire,^23,24^ and their dietary fiber intakes were not much different from the Japanese average.^25^ Fecal samples were collected in tubes and immediately subjected to anaerobic conditions using AnaeroPouches (Mitsubishi Gas Chemical, Tokyo, Japan). Fecal samples were diluted, homogenized with saline (10% w/v), and stored at −80 °C until analysis. Each of the four samples was used individually for the following experiments.

### Fecal Fermentation

Fecal samples were cultured using a pH-controlled multi-channel jar fermenter (Bio Jr.8; ABLE, Tokyo, Japan). Each vessel contained 100 mL of yeast extract, casitone, and fatty acid (YCFA) medium (Table S1) supplemented with a 1% (w/v) carbon source and maintained under anaerobic conditions (100% CO_2_) at 37 °C and pH > 6.0 with constant stirring at 200 rpm during the 24 h fermentation. Each vessel was inoculated with 100 μL of 10× diluted fecal suspension to initiate fermentation. The supplemental carbon sources were inulin (Fuji FF; Fuji Nihon Seito Co., Tokyo, Japan), PHGG (Sunfiber R; Taiyo Kagaku Co., Ltd., Mie, Japan), lactulose (MLC-97; Morinaga Milk Industry Co., Ltd., Tokyo, Japan), raffinose (Nitten Raffinose; Nippon Beet Sugar Manufacturing Co., Ltd., Tokyo, Japan), indigestible dextrin (Fibersol-2; Matsutani Chemical Industry Co., Ltd., Hyogo, Japan), and alginate (ULV-L3; KIMICA Co., Tokyo, Japan). Cultured samples were collected 24 h after the addition of feces. Fecal fermentation with each carbon source was conducted in a single assay for each fecal sample.

### Short-Chain Fatty Acid (SCFA) Analysis

SCFA analysis was performed by the liquid chromatography method.^26^ Each fecal fermentation culture was centrifuged at 8000 × *g* for 10 min at 4 °C, and the supernatant was filtered through a 0.22 μm membrane filter (TORAST Disc NYLON membrane; Shimadzu Co., Kyoto, Japan). The SCFA (formic acid, acetic acid, propionic acid, butyric acid, isobutyric acid, valeric acid, and isovaleric acid) concentrations of each sample were analyzed using HPLC (Shimadzu Organic Acid Analysis System; Shimadzu Co., Kyoto, Japan) with a Shim-pack SCR-102H column (size 300 mm × 8 mm ID, two columns in series) and Shim-pack SCR-102H (50 mm × 6 mm ID) as a guard column. The analysis was performed at a flow rate of 0.8 mL/min and at 45 °C using 5 mmol/L *p*-toluenesulfonic acid as the eluent and a reaction solution containing 5 mmol/L *p*-toluenesulfonic acid, 100 μmol/L EDTA, and 20 mmol/L Bis–Tris.

### Microbiota Analysis

Total DNA extraction, amplification, and sequencing of the V3–V4 region of the bacterial 16S rRNA gene and data analysis were performed as described previously.^22^

### Caco-2/HT29-MTX-E12 Co-Culture Experiments

#### Cell Cultures

Intestinal absorptive cells, Caco-2 (ATCC HTB-37), and intestinal mucin-secreting goblet cells, HT29-MTX-E12 (ECACC 12040401), were separately cultured in Falcon cell culture flasks at 37 °C and 5% CO_2_. The culture medium (DMEM supplemented with 10% FBS, 1% non-essential amino acids, 100 units/mL penicillin, and 100 μg/mL streptomycin) was changed every 2–3 days. Sub-confluent cells were trypsinized using 0.25% trypsin/EDTA and passaged at a ratio of 1:6 twice per week.

#### Co-Culture

A co-culture model of Caco-2 and HT29-MTX-E12 cells was reported to have the permeability features more similar to those of the human intestinal barrier, than the single culture of Caco-2 cells.^27^ The two cell lines were seeded on to the apical surface of 24 well, 0.3 cm^2^, and 0.4 μm pore size Falcon cell culture insert (Corning, NY, USA), at a density of 20,000 cells/well in a 7:3 ratio (Caco-2:HT29-MTX-E12) as described previously.^28^ The culture medium was changed every 2–3 days, and monolayers were used for experiments between Days 21 and 23 post-seeding.

#### Intestinal Barrier Integrity Measurement

The initial transepithelial electrical resistance (TEER) values were obtained using the Millicell-ERS (Millipore, Bedford, MA, USA). The background TEER (insert) was subtracted from the total TEER (cell monolayer + insert) to yield the monolayer resistance and then normalized to the surface area by multiplying with the area of the insert. Cell monolayers with a TEER over 300 Ω cm^2^ were regarded as tight monolayers and used for the experiments. The filtered fermentation supernatant was diluted 1:10 (v/v) with the cell medium and added to the apical side of the cells. The culture medium containing 50 ng/mL TNF-α and 30 ng/mL IFN-γ was added to the basolateral side. After 48 h of incubation, the TEER of each cell monolayer was measured again, and the relative TEER value compared to the initial TEER for each insert was calculated. Experiments were conducted in triplicate for each biological fermentation sample.

### Quantification of Gene Expression

#### RNA Extraction

Total RNA was extracted using the RNeasy Mini Kit (Qiagen, Hilden, Germany) and reverse-transcribed into cDNA using ReverTra Ace qPCR RT Master Mix with gDNA Remover (TOYOBO Co., Ltd., Osaka, Japan).

#### Real-Time PCR

The expression levels of genes (β-actin, *CLDN2*, *CLDN3*, *CLDN4*, and *ZO-1*) in Caco-2/HT29-MTX-E12 co-cultured cells were quantified using TB Green Premix Ex Taq II (Takara Bio Inc., Shiga, Japan). The primer sequences used for the quantification are listed in Table S2. All samples were run on a QuantStudio3 (Applied Biosystems, Waltham, MA, USA). The thermal profile was 95 °C for 30 s, followed by 40 cycles of 95 °C for 3 s and 60 °C for 30 s. β-Actin was used as a housekeeping gene, and the relative gene expression of inflammation-induced cells without fecal fermented samples was used for calibration (control). Experiments were conducted in triplicate for each biological fermentation sample.

### Statistical Analysis

Statistical analyses were performed using JMP version 13 (SAS Institute, Cary, NC, USA). Statistical differences in the relative abundance of the bacterial population, microbial metabolic production, intestinal barrier, and gene expression were compared using Dunnett’s test. The effect of butyric acid on the intestinal barrier function and *CLDN2* expression levels were evaluated using Student’s *t*-test. The correlation between the two parameters was analyzed using the Pearson correlation analysis. The correlation results were not corrected for multiple comparisons. Statistical significance was set at *P* < 0.05.

## Results

### Simulation of the Effects of Various Dietary Fibers and Indigestible Oligosaccharides on Gut Microbiota

First, we used a fecal fermentation system to examine whether the addition of six dietary fibers or indigestible oligosaccharides (inulin, PHGG, lactulose, raffinose, indigestible dextrin, and alginate) alters the gut microbiota of the elderly.

**Figure 1:**
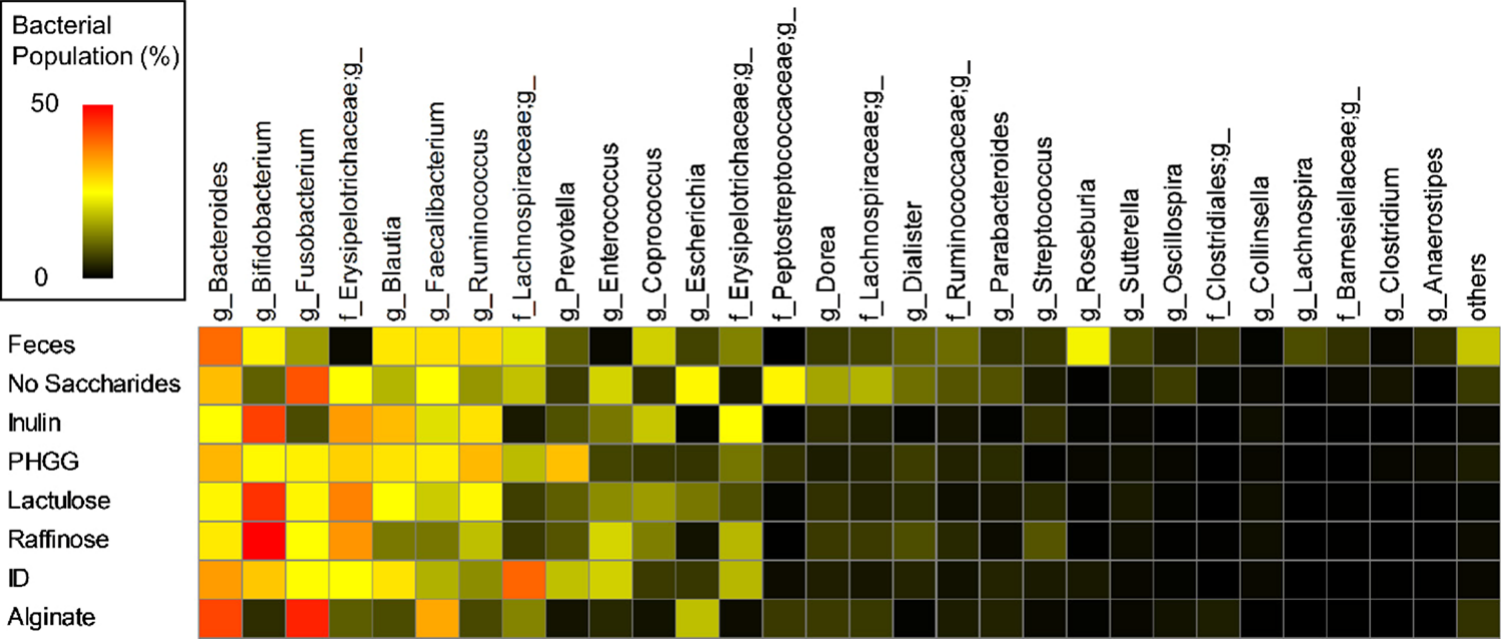
Heatmap showing the top 30 predominant genera in fecal samples and fecal fermented samples. Colors indicate percentages of each bacterial population. Values are expressed as means of the four donors. PHGG, partially hydrolyzed guar gum; ID, indigestible dextrin.

Analysis of the microbiota showed that each prebiotic changed the bacterial composition of the medium after fecal fermentation; furthermore, the bacterial composition differed among the prebiotics (Figure 1). In the absence of saccharides, *Fusobacterium* (27.5 ± 19.4%) and *Peptostreptococcaceae* (6.7 ± 11.6%) showed a high relative abundance, whereas the relative abundance of *Bifidobacterium* was low (1.9 ± 1.0%). Under prebiotic conditions, raffinose, lactulose, and inulin showed an increase in the relative abundance of *Bifidobacterium* (38.0 ± 24.4, 31.8 ± 15.4, and 29.9 ± 20.9%, respectively), and inulin showed the lowest abundance of *Fusobacterium* (1.5 ± 2.3%). PHGG showed the highest relative abundances of *Ruminococcus* (14.3 ± 17.4%) and *Prevotella* (13.5 ± 23.4%). In contrast, alginate showed a different tendency from that of the other fibers and oligosaccharides, with no increase in *Bifidobacterium* (0.9 ± 0.8%), no decrease in *Fusobacterium* (33.7 ± 31.4%), and the highest relative abundance of *Faecalibacterium* (16.4 ± 21.9%), which has been reported as butyric acid-producing bacteria.

### Simulation of Microbial Metabolic Production in the Presence of Dietary Fiber or Indigestible Oligosaccharide

Previous studies have reported that changes in the gut microbiota alter intestinal metabolites.^29^ Therefore, we compared bacterial metabolites (SCFA and LPS) in fecal fermented supernatants without saccharides and in each prebiotic-supplemented condition.

Each metabolite differed between the prebiotic conditions (Table 1). Formic acid was not detected in the absence of saccharides and indigestible dextrin and was significantly increased in the presence of inulin. Acetic acid accounted for the largest percentage of total SCFA production and was significantly increased in all prebiotics except for alginate. Butyric acid showed high variation among the four fecal samples and was not significantly different from the no-saccharide group in any of the prebiotics; however, the mean production of butyric acid increased in all conditions except for indigestible dextrin. With respect to LPS, the mean amount was high in PHGG and alginate, although the difference was not statistically significant. These results indicated that the metabolites in each fecal culture were altered by the addition of different prebiotics.

**Table 1:** Short-Chain Fatty Acid (mM) and Lipopolysaccharide (μg/mL) Concentrations of In Vitro Fecal Fermented Supernatant^a^^,^^b^

	formic acid	acetic acid	propionic acid	butyric acid	isobutyric acid	valeric acid	isovaleric acid	total SCFA	LPS
no saccharides	N.D.	63.5 ± 10.4	14.7 ± 4.0	5.9 ± 2.7	1.5 ± 1.4	0.9 ± 0.1	2.2 ± 2.1	88.7 ± 18.8	3.0 ± 3.7
inulin	26.4 ± 20.8*	114.5 ± 6.9^***^	11.4 ± 1.0	11.1 ± 11.0	0.9 ± 0.1	0.8 ± 0.1	0.9 ± 0.0	166.1 ± 14.2^***^	4.4 ± 3.0
PHGG	10.4 ± 15.8	97.5 ± 13.8^**^	14.5 ± 1.8	8.8 ± 2.6	1.3 ± 0.6	1.0 ± 0.3	1.6 ± 1.0	135.0 ± 2.5^**^	25.3 ± 24.7
lactulose	5.7 ± 5.9	123.3 ± 10.1^***^	10.8 ± 1.9	7.3 ± 9.8	0.8 ± 0.2	0.8 ± 0.1	0.9 ± 0.1	149.6 ± 22.2^***^	6.1 ± 2.8
raffinose	14.9 ± 18.0	102.5 ± 16.9^***^	10.5 ± 1.0	6.9 ± 9.7	0.8 ± 0.1	0.8 ± 0.1	0.8 ± 0.1	137.2 ± 5.8^**^	6.7 ± 1.1
ID	N.D.	90.9 ± 4.1*	14.3 ± 3.6	3.6 ± 1.5	1.1 ± 0.2	0.9 ± 0.1	1.1 ± 0.3	111.9 ± 8.6	3.5 ± 4.0
alginate	2.1 ± 2.6	86.8 ± 16.0	15.3 ± 3.5	10.0 ± 5.2	1.2 ± 0.4	0.9 ± 0.1	2.2 ± 1.1	118.6 ± 21.2	32.3 ± 39.6

a Values are expressed as mean ± SD (four donors). PHGG, partially hydrolyzed guar gum; ID, indigestible dextrin; and LPS, lipopolysaccharide.

b *P < 0.05, **P < 0.01, and ***P < 0.001 indicate significant differences compared to no saccharides (Dunnett’s test).

### Effect of Fecal Culture Supernatants on Intestinal Barrier Function in an Intestinal Inflammation Model

**Figure 2:**
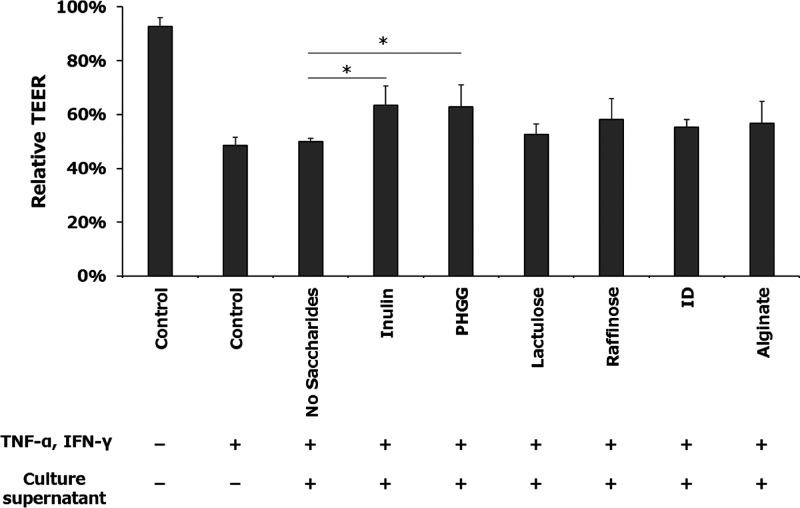
Effect of fecal fermented supernatant on the intestinal inflammation model, Caco-2/HT29-MTX-E12 co-cultured cells with basolateral stressors. Values are expressed as the relative TEER after 48 h compared to the initial TEER. Data are shown as mean ± SD (*n* = 4). *P < 0.05 indicates significant differences compared to no saccharides (Dunnett’s test). Control, no fecal fermented sample (=cell culture medium); PHGG, partially hydrolyzed guar gum; and ID, indigestible dextrin.

Because intestinal metabolites are known to affect the intestinal barrier, we evaluated the effect of fecal culture supernatants of various prebiotics on the inflammation-induced intestinal barrier using intestinal epithelial cells. Caco-2/HT29-MTX-E12 co-cultured cells were used as a model of the intestinal epithelium, and inflammation was induced by the inflammatory cytokines TNF-α and IFN-γ. The culture supernatant without saccharides had little protective effect on the intestinal barrier, whereas the supernatants of dietary fiber or oligosaccharides maintained higher intestinal barrier functions on average compared to that of no saccharides (Figure 2). Among them, inulin and PHGG showed significant protective effects (*P* = 0.030 and 0.039, respectively), indicating that they alter the microbiota and their metabolites in the elderly, which are highly effective in maintaining intestinal barrier function.

### Effect of Fecal Culture Supernatants on Expression Levels of Barrier Function-Related Genes

**Figure 3:**
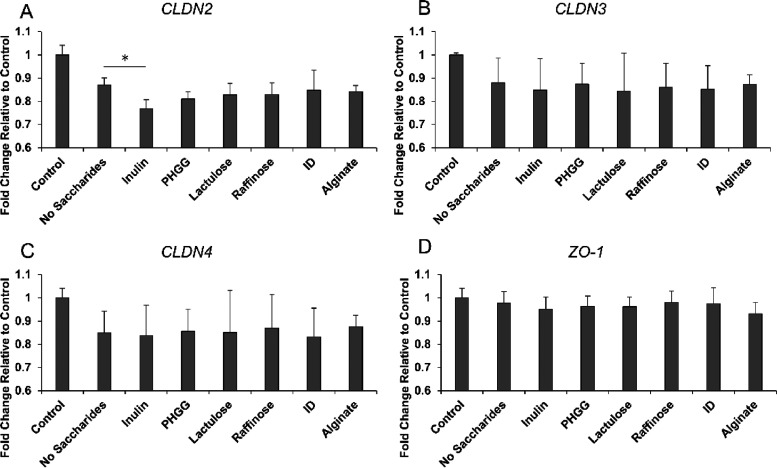
(A–D) Relative gene expression in Caco-2/HT29-MTX-E12 co-cultured cells in response to 48 h exposure to fecal fermented supernatant. Values are expressed as fold change relative to control. Data are shown as mean ± SD (*n* = 4). *P < 0.05 indicates significant differences compared to no saccharides (Dunnett’s test). Control, no fecal fermented sample (=cell culture medium); PHGG, partially hydrolyzed guar gum; and ID, indigestible dextrin.

Various genes are involved in the formation of the intestinal barrier. Therefore, the effect of each fecal culture supernatant on the expression levels of intestinal barrier-related genes in Caco-2/HT29-MTX-E12 co-cultured cells was evaluated. The expression level of the leaky-type channel gene, *CLDN2*, was significantly downregulated in inulin (*p* = 0.034) compared to that in the absence of saccharides (Figure 3). In contrast, no significant changes in the expression levels of *CLDN3*, *CLDN4*, or *ZO-1* were observed for any of the prebiotics. These results suggest that the effect of fecal culture supernatants on the expression levels of *CLDN2* affects intestinal barrier function.

### Evaluation of Factors that Correlate with Intestinal Barrier Function

**Figure 4:**
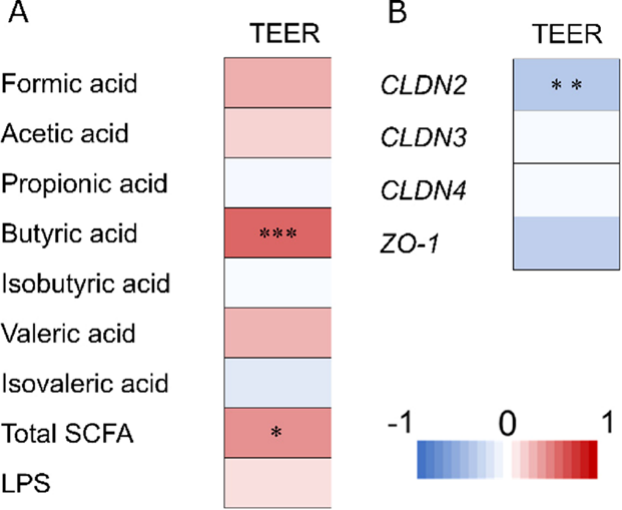
Heatmap showing factors correlated with relative TEER. (A) SCFA and LPS in fecal fermented supernatant. (B) Relative gene expression (*CLDN2*, *CLDN3*, *CLDN4*, and *ZO-1*) of Caco-2/HT29-MTX-E12. Pearson’s correlation analysis was used to test the correlation between two parameters. Correlation coefficients were calculated using data from seven conditions of fecal culture supernatants for each of the four fecal samples. Red represents a positive correlation and blue represents a negative correlation, as shown in the color scale. *P < 0.05; **P < 0.01; and ***P < 0.001.

**Figure 5:**
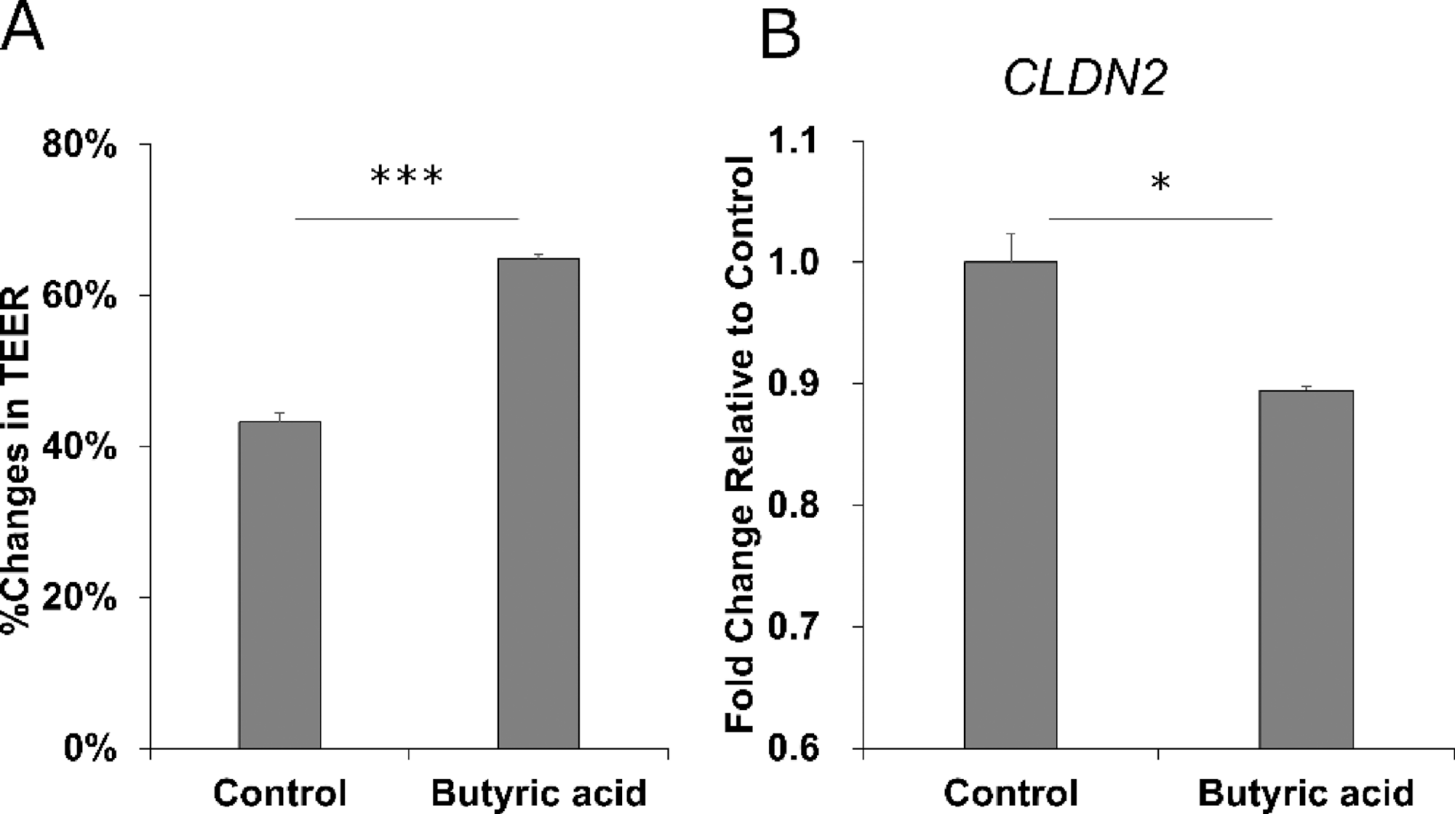
Effect of 2 mM butyric acid on the intestinal inflammation model, Caco-2/HT29-MTX-E12 co-cultured cells with basolateral stressors. (A) Relative TEER after 48 h compared to the initial value. (B) *CLDN2* gene expression in the co-cultured cells. Data are shown as the mean ± SEM (*n* = 3). *P < 0.05 and ***P < 0.001 indicate significant differences by Student’s *t*-test.

In addition, we analyzed the mechanism by which fecal culture supernatants maintain the intestinal barrier function. To determine the factors affecting intestinal barrier function, we evaluated the correlation of barrier function with the amount of some metabolites in the fecal culture supernatant and the expression levels of genes. As for metabolites in the culture supernatant, total SCFA (*r* = 0.44, *p* = 0.019) and butyric acid (*r* = 0.60, *p* = 0.0007) were significantly correlated with the protective effect of the intestinal barrier (Figure 4A). Regarding gene expression levels, *CLDN2* expression levels showed a significant negative correlation (*r* = −0.54, *p* = 0.003) with intestinal barrier function (Figure 4B). To confirm whether butyric acid can affect intestinal barrier function and *CLDN2* gene expression, we added butyric acid to Caco-2/HT29-MTX-E12 co-cultured cells and evaluated barrier function and *CLDN2* expression levels. The culture supernatants of fecal fermentation contained up to approximately 20 mM butyric acid and were diluted 1:10 (v/v) for use in the cell assay. Therefore, the effect of 2 mM butyric acid was evaluated. Compared to the control, the decrease in barrier function was significantly suppressed (*p* = 0.0001; Figure 5A), and the expression level of *CLDN2* was also significantly suppressed (*p* = 0.017; Figure 5B), indicating that the effect of butyric acid on *CLDN2* could be one of the factors retaining the barrier function by the fecal culture supernatant.

## Discussion

We showed that various prebiotics could alter the fecal microbiota of the elderly in vitro, and the bacterial metabolites produced by the microbiota grown in the presence of inulin and PHGG are particularly effective in maintaining the intestinal barrier. We also demonstrated that among the metabolites of intestinal bacteria, butyric acid, in particular, contributes to intestinal barrier protection, and the suppression of *CLDN2* expression can be one of the factors involved in this protection.

Some SCFAs, such as acetic acid, propionic acid, and butyric acid, help enhance intestinal barrier function, and butyric acid has a particularly strong effect.^30^ In this study, the production of total SCFAs and, in particular, butyric acid, showed a high correlation with barrier function, suggesting that they were the main factors contributing to barrier function. However, although butyric acid was highly correlated with barrier function, butyric acid production was not significantly increased in any of the prebiotics, including inulin and PHGG, which may be due to the large variation in butyric acid production among the fecal samples. Alginate, which showed a relatively high average butyric acid production, and lactulose, which showed a significant increase in total SCFAs, showed no protective effect on barrier function. Thus, the amount of total SCFA and butyric acid produced alone does not fully explain the protective effect of the intestinal barrier. Several kinds of bacterial metabolites were found in the culture supernatant, and these metabolites probably affect barrier function. LPS has been reported to impair the barrier function.^31^ The mean amount of LPS in the fermented supernatant was the highest in alginate, which could have disturbed the enhancement in the barrier function even though butyric acid production was high (Table 1). However, LPS levels did not correlate with barrier function, suggesting that LPS had little effect on barrier function. Formic acid production was significantly high in inulin but did not significantly correlate with barrier function (*r* = 0.33, *p* = 0.089) or *CLDN2* gene expression (*r* = −0.33, *p* = 0.087). The physiological effects of formic acid produced by gut bacteria have hardly been studied because they are metabolized to other metabolites and their concentrations in the intestinal lumen of adults are low. A recent study reported that formic acid is produced by *Bifidobacterium* and is accumulated in the intestinal lumen of breastfed infants.^32^ Formic acid may possibly play a functional role in intestinal health, and the role is expected to be verified. Although not measured in this study, ethanol and acetaldehyde produced by gut microbiota are known to have a negative effect on barrier function,^33,34^ and glutamine and tryptophan, whose concentrations are altered because they are utilized as the carbon source of the microbiota, are known to have a positive effect on barrier function.^35,36^ These combined factors could have affected the current results.

The mechanism by which inflammation-induced barrier dysfunction is maintained by a mixture of intestinal bacterial metabolites, such as fecal culture supernatants, has not been clarified in detail. Butyric acid has been reported to inhibit the expression of the tight junction-related gene, *CLDN2*, in intestinal epithelial-like cells.^37^ Among tight junction proteins, claudin-2 has the unique property of serving as a pore-forming protein in tight junction, and its upregulation induces disruption of the barrier.^37^ Consistent with the report, butyric acid maintained the barrier function; furthermore, it suppressed the *CLDN2* expression level in the co-cultured cells. This suggests that butyric acid in the fecal culture supernatant with inulin may regulate the barrier function of the elderly intestinal epithelium through *CLDN2* downregulation.

In microbiota analysis, the effects of prebiotics on altering the microbiota of the elderly differed depending on the type of prebiotic. The microbiota does not seem to be similar, even when PHGG and inulin are being compared, which can both effectively protect the intestinal barrier. In the case of inulin, *Bifidobacterium* and *Erysipelotrichaceae* had relatively high abundance, whereas *Bacteroides* and *Lachnospiraceae* had low abundance, which seems similar to the cases of lactulose and raffinose rather than PHGG. Despite the differences in microbiota, PHGG also showed a high barrier protective effect, suggesting that the different bacterial groups affected the intestinal barrier via butyric acid and other factors between inulin and PHGG. In inulin, the abundances of *Bifidobacterium*, acetic acid-producing bacteria, and *Coprococcus*, butyric acid-producing bacteria, are high,^38,39^ whereas the mean abundance of *Fusobacterium*, which is considered to be involved in colitis,^40^ is low, which may have contributed to the barrier function. In contrast, in PHGG, the mean abundances of butyric acid-producing bacteria such as *Faecalibacterium* and *Ruminococcus*(38,39,41) tended to be higher than those of the others, which may have contributed to intestinal barrier protection. As mentioned before, the decrease in *Bifidobacterium* and the increase in *Fusobacterium* have been identified as issues in the elderly.^1,42^ Therefore, considering the effects of prebiotics other than those on the intestinal barrier, inulin may be more suitable for the elderly.

Several studies, as well as this study, evaluated the effects of in vitro fecal fermented supernatants of several dietary fibers on the intestinal barrier.^6,43^ In a study on the analysis of feces of three healthy young Belgians (25–31 years old), arabinoxylo-oligosaccharides and inulin levels were evaluated, and a significant enhancement of barrier function was observed with inulin compared to the blank control.^43^ The study using feces from three healthy Americans (22–26 years old) evaluated inulin-based, oat β-glucan-based, and xylo-oligosaccharide-based samples, and found that all of them significantly suppressed the increase in intestinal permeability caused by basolateral stressors.^6^ In addition, one study reported that 20 healthy young Italians (average age 18.8 years old) with long-term intake of inulin mixed in pasta showed improvement in barrier function characterized by a decrease in lactulose permeability and blood zonulin.^18^ These studies have shown that inulin can effectively enhance the intestinal barrier of young people. This study indicated that inulin also alters the gut microbiota of the elderly, and its metabolites have a relatively high protective effect on the leaky barrier, suggesting that inulin may affect a wide range of ages.

This study had several limitations. First, we did not consider the possibility that prebiotics remaining in the medium directly affected the intestinal barrier. However, the contribution of the remaining prebiotics itself was thought to be little because a previous study showed that the protective effect of prebiotics on the intestinal barrier is attributed mainly to intestinal bacterial metabolites.^6^ Second, the protective effect of prebiotics was evaluated only in vitro. Third, the protein levels of tight junction proteins were not confirmed. Finally, the small number of fecal samples is also a limitation.

In this study, the intestinal barrier function was protected from inflammation-induced damage by the fecal fermented supernatant of inulin and PHGG from the elderly. In addition, we showed that the regulation of *CLDN2* via butyric acid may be an important factor in the protection of the intestinal barrier. Among the elderly, the intake of prebiotics such as inulin and PHGG may be expected to maintain intestinal barrier function and prevent bacterial translocation and the influx of toxins into the body. Although the main focus of this study was to protect the intestinal barrier, it is also necessary to determine useful prebiotics for the elderly, considering other functions of prebiotics in real life. Because most research on prebiotics has been conducted on healthy young people and the types of prebiotics affecting health might differ between young and elderly people, further research on the elderly, including effects other than those on the intestinal barrier, is required to improve the health of the elderly.

## Supporting Information Available

Compositions of YCFA media; primer sequences for quantification of β-actin, *CLDN2*, *CLDN3*, *CLDN4*, and *ZO-1* by qPCR (PDF)
