# Supplementary material for: Fermentation Supernatant of Elderly Feces with Inulin and Partially Hydrolyzed Guar Gum Maintains the Barrier of Inflammation-Induced Caco-2/HT29-MTX-E12 Co-Cultured Cells
Source: J Agric Food Chem. 2023 Jan 9;71(3):1510–7. doi: 10.1021/acs.jafc.2c06232 (PMC9880993; doi:10.1021/acs.jafc.2c06232)
Supplement: Supplementary file 1 — jf2c06232_si_001.pdf [file jf2c06232_si_001.pdf]

■Supporting Information

Table S1. Compositions of YCFA medium.

Compounds	Formula	Concentration(/L)	Manufacturer
Bacto™ Casitone		10.0 g	Becton Dickinson and Company
Bacto™ Yeast Extract		2.5 g	Becton Dickinson and Company
Sodium hydrogen carbonate	$NaHCO_3$	4.0 g	FUJIFILM Wako Pure Chemical Corp.
Hemin	$C_{34}H_{32}ClFeN_4O_4$	0.01 g	HYDRUS CHEMICAL INC.
Resazurin	$C_{12}H_7NO_4$	1 mg	TOKYO CHEMICAL INDUSTRY CO.,LTD.
Dipotassium hydrogen phosphate	K_2HPO_4	0.45 g	FUJIFILM Wako Pure Chemical Corp.
Potassium dihydrogen phosphate	KH_2PO_4	0.45 g	FUJIFILM Wako Pure Chemical Corp.
Ammonium sulfate	$(NH_4)_2SO_4$	0.9 g	KOKUSAN CHEMICAL Co. Ltd
Sodium chloride	NaCl	0.9 g	FUJIFILM Wako Pure Chemical Corp.
Magnesium sulfate	$MgSO_4$	0.045 g	KOKUSAN CHEMICAL Co. Ltd
Calcium chloride dihydrate	$CaCl_2 \cdot {}_2H_2O$	0.09 g	FUJIFILM Wako Pure Chemical Corp.
L-cysteine	$C_3H_7NO_2S$	1.445 g	Kanto Chemical Co., Inc.
Biotin	$C_{10}H_{16}N_2O_3S$	0.01 mg	NACALAI TESQUE, INC
Cobalamin	$C_{63}H_{88}CoN_{14}O_{14}P$	0.01 mg	NACALAI TESQUE, INC
p-Aminobenzoic acid	$C_7H_7NO_2$	0.03 mg	NACALAI TESQUE, INC
Folic acid	$C_19H_19N_7O_6$	0.05 mg	FUJIFILM Wako Pure Chemical Corp.

Compounds	Formula	Concentration(/L)	Manufacturer
Pyridoxamine	$C_8H_{12}N_2O_2$	0.15 mg	SIGMA ALDRICH
Thiamine Hydrochloride	$C_{12}H_{18}Cl_2N_4OS$	0.05 mg	SIGMA ALDRICH
Riboflavin	$C_{17}H_20N_4O_6$	0.05 mg	FUJIFILM Wako Pure Chemical Corp.
Acetic acid	$C_2H_4O_2$	35 mmol	KOKUSAN CHEMICAL Co. Ltd
Propionic acid	$C_3H_6O_2$	9 mmol	FUJIFILM Wako Pure Chemical Corp.
n-valeric acid	$C_5H_{10}O_2$	1 mmol	FUJIFILM Wako Pure Chemical Corp.
Iso-valeric acid	$C_5H_{10}O_2$	1 mmol	FUJIFILM Wako Pure Chemical Corp.
Iso-butyric acid	$C_4H_8O_2$	1 mmol	FUJIFILM Wako Pure Chemical Corp.

Table S2. Primer Sequences for Quantification of *β-actin*, *CLDN2*, *CLDN3*, *CLDN4*, *ZO-1* by qPCR.

Primer	Forward (5' to 3')	Reverse (5' to 3')
β-actin[1]	TGGCACCCAGCACAATGAA	CTAAGTCATAGTCCGCCTAGAAGCA
CLDN2[2]	TACTCACCACTGGTGCCTGA	GAGAGCTCCTTGTGGCAAGA
CLDN3[1]	ACATCATCACGTCGCAGAACATC	AGTGCCAGCAGCGAGTCGTA
CLDN4[3]	TATGGATGAACTGCGTGGTG	CCACGATGATGCTGATGATG
ZO-1[1]	CGGGACTGTTGGTATTGGCTAGA	GGCCAGGGCCATAGTAAAGTTTG

References

(1) Park, H. Y.; Kunitake, Y.; Hirasaki, N.; Tanaka, M.; Matsui, T., Theaflavins enhance intestinal barrier of Caco-2 Cell monolayers through the expression of AMP-activated protein kinase-mediated Occludin, Claudin-1, and ZO-1. *Bioscience, biotechnology, and biochemistry* **2015,** *79* (1), 130-137.
(2) Vila, L.; García-Rodríguez, A.; Cortés, C.; Marcos, R.; Hernández, A., Assessing the effects of silver nanoparticles on monolayers of differentiated Caco-2 cells, as a model of intestinal barrier. *Food and chemical toxicology : an international journal published for the British Industrial Biological Research Association* **2018,** *116* (Pt B), 1-10.
(3) Mengxuan, S.; Yunshuang, Y.; Chen, M.; Li, D.; Fang, C., Pasteurized Akkermansia muciniphila Ameliorate the LPS-Induced Intestinal Barrier Dysfunction via Modulating AMPK and NF-κB through TLR2 in Caco-2 Cells. *Nutrients* **2022**, 14, 764.
